# Molecular Imprinted Polymers on Microneedle Arrays for Point of Care Transdermal Sampling and Sensing of Inflammatory Biomarkers

**DOI:** 10.1021/acsomega.2c04789

**Published:** 2022-10-24

**Authors:** Daniela Oliveira, Barbara P Correia, Sanjiv Sharma, Felismina Teixeira Coelho Moreira

**Affiliations:** † BioMark Sensor Research, ISEP, School of Engineering, Polytechnic Institute, Porto 4200-072, Portugal; ‡ CEB, Centre of Biological Engineering, Minho University, Braga 4704-553, Portugal; § LABBELS - Associate Laboratory, Braga, 4806-909 Guimarães, Portugal; ∥ Department of Biomedical Engineering, Faculty of Science and Engineering, Swansea University, Swansea SA1 8EN, U.K.

## Abstract

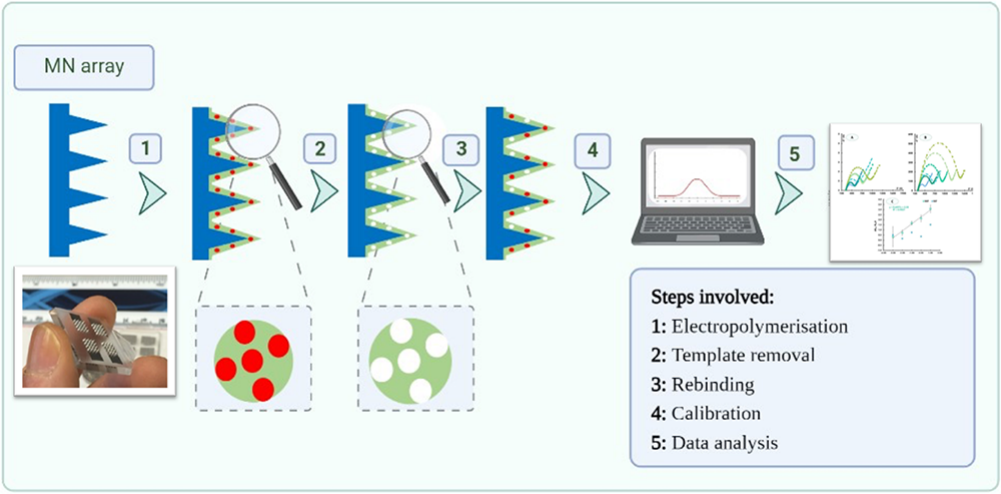

The skin interstitial fluid (ISF) contains biomarkers that complement other biofluids such as blood, sweat, saliva, and urine. It can be sampled in a minimally invasive manner and used either for point of care testing or real time, continuous monitoring of analytes, the latter using microneedle arrays. The analytes present in the skin ISF are indicative of both systemic and local (i.e., skin) physiology. In this paper, we describe combining microneedle technology with molecularly imprinted polymers to demonstrate the potential of transdermal electrochemical sensing. The molecularly imprinted polymer employed here is easy to produce; it can be thought of as plastic antibody. Its synthesis is scalable, and the resulting sensor has a short measurement time (6 min), with high accuracy and a low limit of detection. It provides the requisite specificity to detect the proinflammatory cytokine IL-6. IL-6 is present in the skin ISF with other cytokines and is implicated in many clinical states including neurodegenerative diseases and fatal pneumonia from SARSCoV 2. The ability to mass produce microneedle arrays and plastic antibodies will allow for low-cost transdermal sensing devices. The transdermal sensors were able to detect IL-6 at concentrations as low as 1 pg/mL in artificial skin ISF, indicating its utility for routine point of care, bloodless measurements in simpler settings, worldwide.

## 1 Introduction

The skin is the largest organ in the body; the skin interstitial fluid (ISF) with its volume larger than blood offers a sample matrix with a plethora of analytes of clinical significance. Skin ISF is an ultrafiltrate of plasma and therefore shows compositional similarities with biofluids. It is also a medium of transport of nutrients and metabolic end products between cells and capillary blood, which makes it a treasure trove of biomarkers. The analytes of clinical interest include metabolites, drugs, and biomarkers implicated in various diseases that offer valuable insight on systemic and dermatological physiology. The skin also shares an ectodermal origin with other organs such as the brain, therefore making it plausible to look there for biomarkers of pathological alterations in the brain.^1^ The skin has been referred to as a window to the body and, as a vital clinical sample matrix, has the potential to facilitate early diagnosis and screening in simple settings, employing low-cost diagnostic devices.

Techniques such as microdialysis and suction blister formation have been used for sampling skin ISF. However, these techniques are invasive, painful, time consuming, and do not yield real-time information. Moreover, they result in local inflammation, therefore skewing the analysis of markers for this condition such as IL-6. Microneedle arrays originally introduced for transdermal drug and intradermal vaccine delivery have become popular over the last decade for transdermal sensing by virtue of their ability to sample the skin ISF in a minimally invasive and pain-free manner. Typically ranging from 0.5 to 1 mm in height and with tip dimensions ranging from 10–40 μm, they offer a platform for wearable molecular sensing of analytes present in the skin ISF. Biomarkers such as metabolites including glucose,^2^ lactate,^3^ and alcohol^4^ have been monitored continuously in real time using microneedle-based wearable molecular biosensors. Similarly, therapeutic drug levels have been monitored over several hours, sufficient to follow their pharmacodynamics and pharmacokinetics in this compartment.^5^ Cytokines such as interleukins have been monitored in a point of care format using immunodiagnostic microneedle arrays.^6^

Cytokines are a family of signaling proteins ranging in molecular mass between 6 and 80 kDa. They are associated with many vital functions including directing the innate immune response in mammalian systems and have been implicated in various pathologies ranging from neurodegenerative diseases to viral infections.^7−9^ Interleukin 6 (IL-6) is a proinflammatory cytokine that can be produced by virtually every nucleated cell type. During inflammation, it is produced along with other proinflammatory cytokines such as interleukin-1β (IL-1β), IL-4, IL-10, macrophage chemoattractant protein-1 (MCP-1), and tumor necrosis factor-α (TNF-α). These cytokines can be sampled from the skin under various conditions using microdialysis probes.^10,11^ The disadvantage with the microdialysis sampling approach is that cytokines are not recovered with 100% efficiency, and thus calibration becomes a challenge.^12^

So far, the microneedle-based continuous and point of care diagnostic devices have mainly employed enzymes^2−5^ or aptamers^13^ as bioreceptor elements for the construction of biosensing devices. Molecular imprinting is a technique used to synthesize highly cross-linked polymers capable of selective molecular recognition. They have been successfully employed as plastic antibodies for several healthcare applications targeting biomarkers of several infectious, cardiovascular, and neurological diseases.^14−19^ Compared to protein- or nucleic acid-based molecular receptors, their advantages include reusability, biosafety, and biocompatibility, which make them a suitable platform for potential clinical blood purification applications.^20^ From an electrochemical biosensing perspective, MIPs offer features such as ease of biosensors construction, precise specificity, sensitivity, and the required dynamic range for biomarkers of interest.

The ease of mass fabrication of microneedle arrays using scalable injection molding technology^21^ makes the combination of microneedle arrays and molecular imprinted polymer/artificial antibody technology an ideal platform offering low-cost transdermal sensing devices. MIPs can easily be coated on out-of-plane microneedle arrays and employed as plastic antibodies. Here, we describe a significant new electrochemical sensing concept born out of the marriage of microneedle arrays and molecular imprinted polymers for point of care testing for the inflammatory cytokine IL-6 in artificial dermal ISF.

## 2 Materials and Methods

### 2.1 Materials and Reagents

The following reagents, potassium hexacyanoferrate(III) (K_3_[Fe(CN)_6_]), potassium hexacyanoferrate(II) (K_4_[Fe(CN)_6_]) trihydrate, and sucrose, were obtained from Riedel-de Häen; potassium chloride (KCl) was obtained from Carlo Erba; phosphate buffered saline (PBS, 0.01 M, pH 7.4) solution and magnesium sulfate (Mg_2_SO_4_) were obtained from Panreac; proteinase K and HEPES buffer were obtained from Sigma-Aldrich; 3-aminophenylboronic acid monohydrate 98% was obtained from Acros Organics; sodium dihydrogen phosphate dihydrate was obtained from Scharlau; calcium chloride (CaCl_2_) was obtained from Merck; glucose was obtained from Alfa Aesar; potassium chloride and sodium chloride were obtained from Normapur; and interleukin 6 (IL-6), 10 μg/mL, was obtained from Abcam. Electrical wires and insulating varnish were obtained from RS Components. All electrochemical measurements were done employing a potentiostat/galvanostat (Metrohm Autolab), equipped with an impedimetric module and controlled by NOVA 2.1.5 software.

### 2.2 Polycarbonate Microneedle Array (MNA) Structures

The polycarbonate MNAs were fabricated by injection molding of polycarbonate using a previously established protocol.^21^ Each polycarbonate structure had four regions, with each region comprising an array of 16 (4 × 4) microneedles. Each pyramidal microneedle is 1000 μm in height, with a 600 μm square base and a tip diameter of 20 μm. The pitch between the microneedles is 1200 μm. The microneedle structures reported here have been thoroughly characterized using scanning electrochemical microscopy (SEM) and optical coherence tomography to show insertion in human skin.^2^

The poly(carbonate) arrays were metallized. Four separate areas of microneedles were first sputter-coated with an initial seed layer of chrome (110 nm). Following this, three sections (two forming working electrodes and one forming counter electrode) were coated with 150 nm of platinum by e-beam evaporation and the final section (forming the reference electrode) was coated with 150 nm of silver by e-beam evaporation. The metallized arrays were wired to metal contacts using silver epoxy (RS components).

To create the Ag|AgCl reference electrode, the silver pad of the array was chloridized with ferric chloride (0.1 M solution) (10 μL) for 30 s. The electrode was washed thoroughly with deionized water and dried with a stream of compressed air. Each electrode was tested by cyclic voltammetry in a solution of 5 mM potassium hexacyanoferrate(III)/(II).

### 2.3 Sensor Fabrication

**Figure 1:**
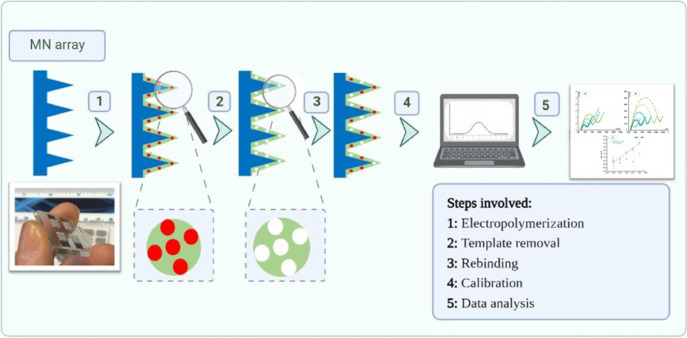
Schematic representation of the construction of MIP-based sensing devices on MNA.

As illustrated in Figure 1, the molecular imprinted polymers (MIPs) were fabricated in two steps involving electropolymerization of the monomer 3-aminophenylboronic acid (APBA) followed by subsequent protein removal. In the first step, a solution consisting of APBA (5 mM) and IL-6 (10 μg/mL) in PBS buffer (pH = 7.4) was used to assemble the MIP layer by electropolymerization on one of the MNAs and designated as the MIP array. This was carried out by CV (−0.2 to 1.0 V, 15 cycles, 0.02 V/s).

Another MNA was modified to an NIP (nonmolecularly imprinted polymers) array, employing a solution consisting of just the 3-aminophenylboronic acid (5 mM) in PBS buffer (pH = 7.4), without the protein.

Subsequently, the polymeric films (both MIP and NIP arrays) were washed with ultrapure water and incubated with 20 μL of proteinase K (500 μg/mL in PBS buffer) overnight at 40C. The sensors were subject to an electrochemical pretreatment by cycling the potential (−0.2 to 1.0 V, 15 cycles, 0.02 V/s) in PBS buffer (pH = 7.4). This procedure removes IL-6 fragments adsorbed on the polymer matrix following proteolytic digestion, thereby yielding cavities that are complementary to the IL-6 proteins in size and shape.

### 2.4 Sensor Calibration in Artificial Interstitial Fluid

To monitor the response in physiological conditions, we employed artificial interstitial fluid (ISF). Artificial ISF was prepared by mixing 2.5 mM CaCl_2_, 5.5 mM glucose, 10 mM HEPES, 3.5 mM KCl, 0.7 mM MgSO_4_, 123 mM NaCl, 1.5 mM NaH_2_PO_4_, and 7.4 mM sucrose. The pH was adjusted to pH 7.4.^22^ Before calibration, the MNA designated as MIP and NIP were incubated with the different concentrations of IL-6 in buffered artificial interstitial fluid, for a period of 20 min.

### 2.5 Eletrochemical Assays

All cyclic voltammetry (CV) and electrical impedance spectroscopy (EIS) measurements were conducted in triplicate employing a redox probe comprising 5.0 mM [Fe(CN)_6_]^3–^ /[Fe(CN)_6_]^4–^, prepared in PBS buffer (pH 7.4) and artificial ISF (pH 7.4). In CV assays, potentials were scanned from −0.4 to +0.7 V, at 50 mV/s. For the EIS measurements, a potential of +0.15 V was set, using a sinusoidal potential perturbation of 0.01 V amplitude with 50 frequencies logarithmically distributed over the range from 50 Hz to 10 kHz. All EIS data was fitted to a Randles equivalent circuit. Calibration curves were constructed by EIS using IL-6 standard solutions prepared in artificial ISF (pH 7.4) ranging from 1 pg/mL to 100 ng/mL.

## 3 Results and Discussion

### 3.1 Molecular Imprinting of IL-6 Cytokines on MNA

Molecular imprinting was performed by electropolymerization of the APBA monomer using IL-6 as a template molecule by CV. The whole process consisted of two distinct phases: (1) imprinting by APBA mixed with IL-6 (bulk solution), which formed a thin film on the surface of the (MNA); and (2) removal of IL-6 from the APBA film by protease activity (Figure 1). All these phases resulted in changes in the electron transfer properties of the receptor surface and were observed in the EIS and CV assays.

Many electrosynthesized polymers have been employed as molecular imprinting materials,^23^ but 3-APBA-based polymer films have several advantages, including easy control of polymer thickness due to self-limiting growth and a simple regeneration process after use.^24^ In addition, since IL-6 is a glycosylated cytokine, it is compatible with the boronic acid functional group in APBA. This is advantageous because boronic acid can covalently react with cis-diols to form five- or six-membered cyclic ester in an alkaline aqueous solution, which dissociates when the medium changes to an acidic pH. This remarkable chemistry makes boronic acids an interesting ligand for the many applications in sensing, separation, and self-assembly.^25^

**Figure 2:**
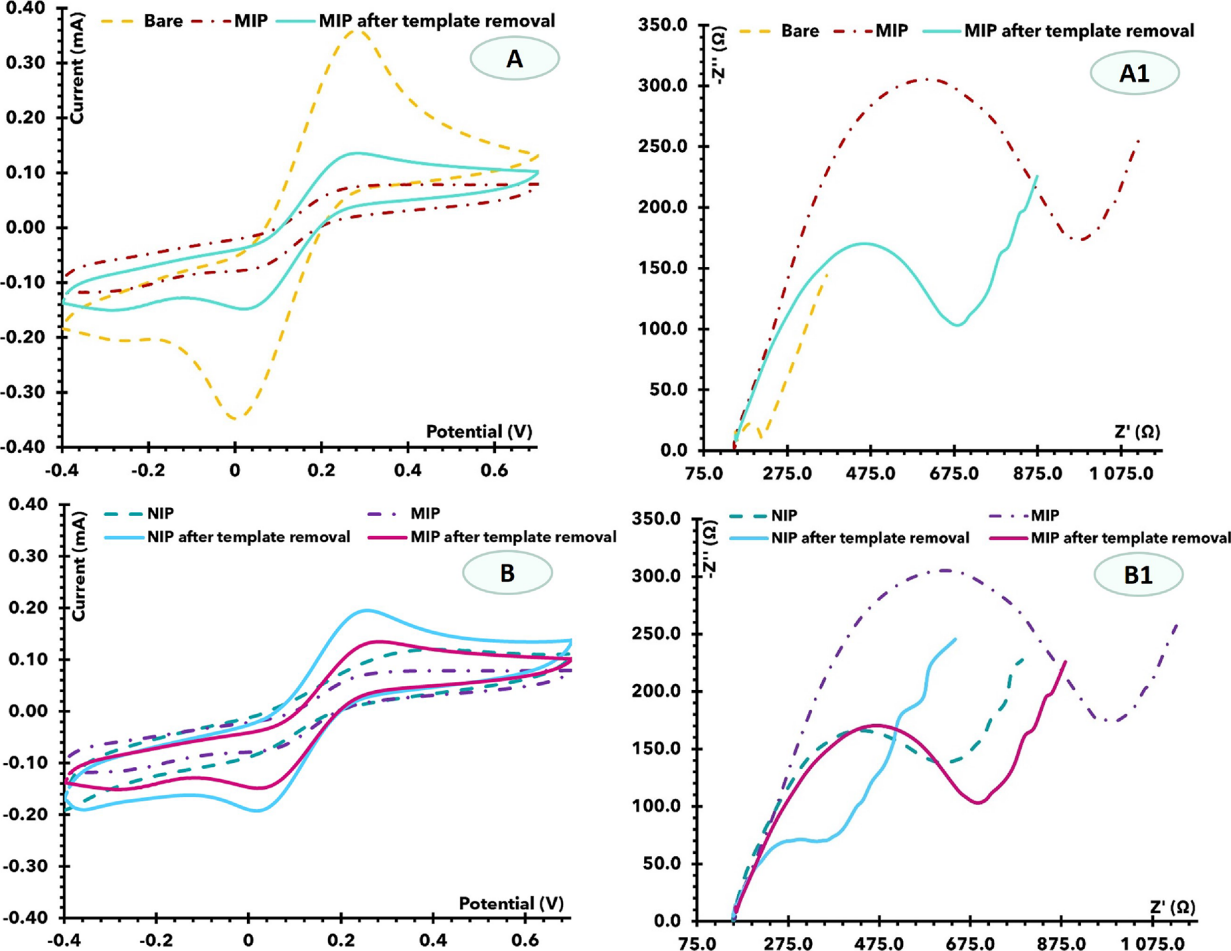
CV (A, B) and EIS (A1, B1) measurements of the devices at the several stages of the biosensor assembly, including the bare MNA, electropolymerization process, and removal of the target molecule. Images (A) and (A1) correspond to the formation of the MIP. In (B) and (B1), we observed the comparison of MIP and NIP.

In general, the EIS data obtained with the polymer film were consistent with the formation of an insulating layer. The typically low Rct of platinum increased very sharply, reaching values of *Z*″ of more than 1000 Ω after the polymerization step with the included protein (MNA/poly 3-APBA + IL-6) (Figure 2A1). Overall, in this model, the impedance of a faradaic reaction consists of an active charge transfer resistance, Rct, and a specific electrochemical diffusion element, ZW, also called the Warburg element (ZW = AW/(*j*ω)0.5, where AW is the Warburg coefficient, *j* is the imaginary unit, and ω is the angular frequency).

As shown in Figure 2B1, an enlargement of the semicircle was observed after NIP polymerization, proving the presence of an insulating polymer on the MN surface. However, compared to the MIP sensor, its resistance is much higher. This is attributed to the presence of the protein in the polymer matrix of the MIP, which provides additional resistance for electron transfer. The measurements from CV agree with the EIS measurements (Figure 2A,B). After MIP polymerization, a decrease in peak current and an increase in peak separation were observed compared to the bare MNs. The CV data for the NIP material agreed with the EIS. Overall, a lower peak current and higher peak spacing were observed for MIP compared to NIP, which can be attributed to the presence of the protein in the polymer matrix.

### 3.2 Protein Removal

The removal of the template was due to the action of proteinase K. The enzymes are effective and act under mild conditions so that the polymeric network is not significantly altered. The MNA/poly 3-APBA polymer film with IL-6 was therefore incubated overnight with 500 μg/mL proteinase K at 4 °C. Proteinase K is a highly active and stable protease with low specificity with respect to the peptide bond environment and high efficiency in cleaving the amide bond. The resulting peptide fragments of IL-6, together with the free enzyme adsorbed on the MIP surface after enzymatic digestion, were removed by CV in PBS buffer (pH = 7.4).

After removal of the template from the MIP sensor, a decrease in the semicircle diameter was observed in the Nyquist diagram. This behavior is likely due to the absence of the protein in the polymer matrix and the presence of voids in the shape of the protein. The data from CV agree with the EIS measurements. After removal of the template, a decrease in peak spacing and an increase in peak current were observed, indicating that electron transfer was enhanced by the absence of the protein. A slight decrease in the diameter of the semicircle was observed in the EIS data from the NIP sensor. This could be due to the CV treatment after proteinase K incubation, which could remove some unreacted monomers from the NIP surface. The data from CV confirms these results. A slight increase in peak current was observed after this treatment.

### 3.3 Analytical Performance of the Sensors in Artificial Interstitial Fluids

**Figure 3:**
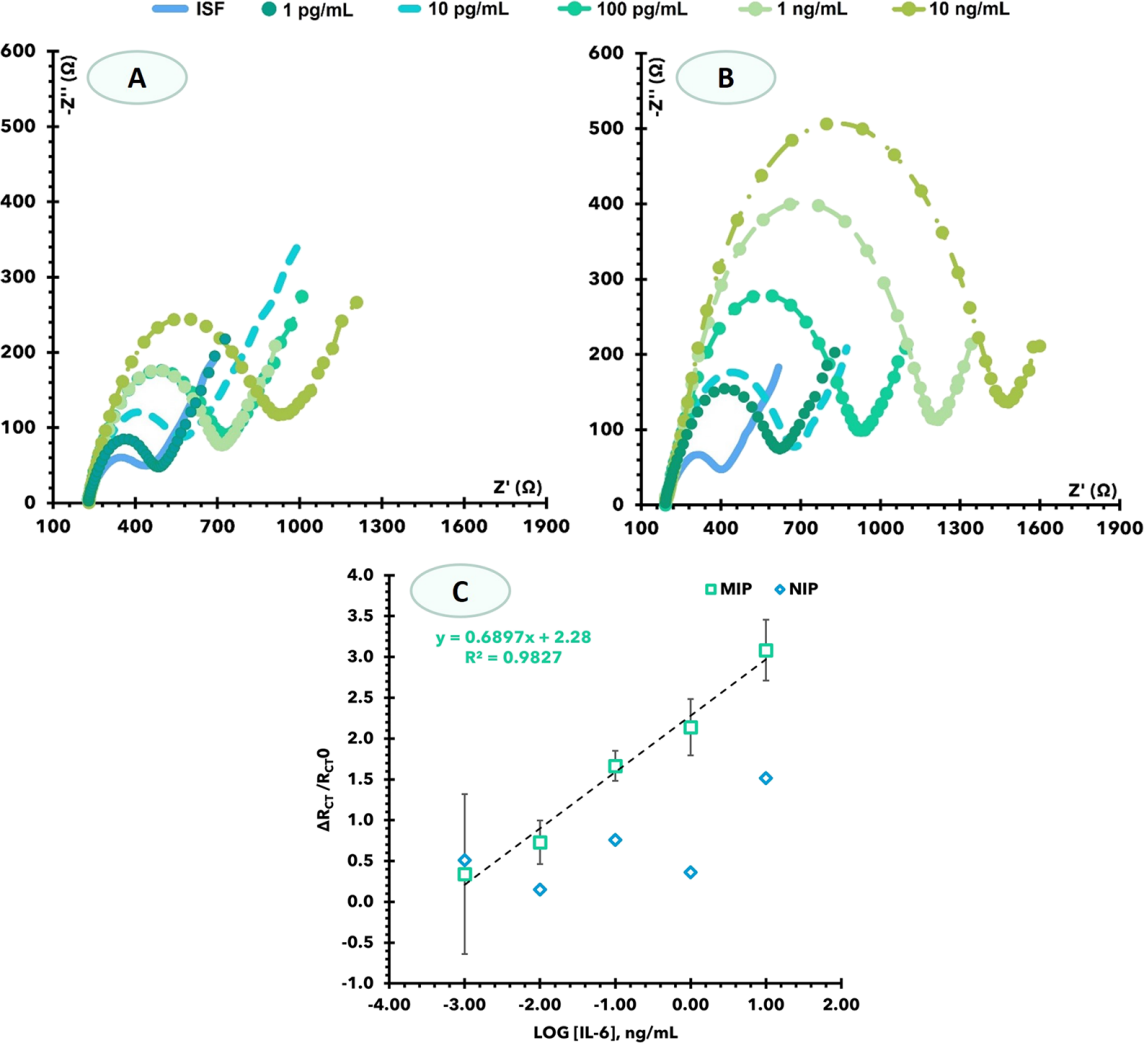
Calibration of the IL-6 biosensor in interstitial fluid NIP (A) and MIP (B) Nyquist plots; (C) corresponding calibration curve at 5.0 mM [Fe(CN)6]^3−^ and 5.0 mM [Fe(CN)6]^4−^, in standard solution-prepared interstitial fluid in using relative Rct data.

Access to human interstitial fluid requires employing painful procedures such as microdialysis or suction blisters performed after research ethical approvals. Employing these painful methods result in inflammation at the site of extraction and so would be expected to increase IL-6 (to an unknown extent). In the absence of these resources, artificial interstitial fluid as described in Section 2 was prepared to assess the analytical performance of the sensors in near physiological conditions. The results obtained are presented in Figure 3. Artificial ISF spiked with varying concentrations of IL-6 showed good features in terms of lower concentration of the linear concentration range. All assays were performed with three repeated measurements.

EIS semilogarithmic calibration curves were recorded for MN-MIP and MNA-NIP electrodes with IL-6 concentration, ranging from 1.0 pg/mL to 10 ng/mL with the presence of IL-6 in the solution concomitantly increasing the resistance on the MNA surface (Figure 3).

With regards to the EIS data obtained from MIP-MNA, the Rct values in the Nyquist plots increased linearly with the increase of the logarithm of IL-6 concentration after 1.0 pg/mL (Figure 3). The slope average was 0.69 Ω/decade [IL-6, pg/mL], and the squared correlation coefficient was >0.9827. The LLD was 1.0 pg/mL. The NIP-MNA sensor does not display linear behavior in the entire range of the calibration curve.

## 4 Conclusions

The effective combination of MNA array platforms for sampling and diagnostics with molecular imprinting technology in an analytical point of care (PoC) device provides a promising tool for direct electrical detection of proteins in the skin compartment in a minimally invasive manner. The simple translation of screen-printed protocols for MIP to MNA-based MIPs will lead to a plethora of transdermal diagnostic applications. This will include various pathologies ranging from neurodegenerative diseases to viral infections. The various components of the skin make it an immunologic organ; therefore, most of the cytokine biomarkers of clinical significance are present in the skin compartment. Single cytokine measurements such as IL-6 are of limited diagnostic use as the innate immune system is relatively nonspecific. A panel approach is therefore necessary, one for which MNAs are well suited. In general, the transdermal sensor presented here showed simplicity in designing, short measuring time, high accuracy, and low limit of detection. This approach seems a successful tool for screening of inflammatory biomarkers in point of care testing wherein the skin acts as a window to the body.

## Abbreviations

MNA: microneedle array
ISF: interstitial fluid
MIP: molecular imprinted polymer
NIP: nonimprinted polymer
